# Clinical and echocardiographic predictors of postoperative atrial fibrillation in lung surgery: the role of left atrial remodelling

**DOI:** 10.1007/s11739-025-03930-6

**Published:** 2025-04-08

**Authors:** Valentina Scheggi, Alberto Salvicchi, Silvia Menale, Jacopo Giovacchini, Stefano Fumagalli, Emanuele Santamaria, Giulia Spanalatte, Rossella Marcucci, Luca Voltolini, Niccolò Marchionni

**Affiliations:** 1 Division of Cardiovascular and Perioperative Medicine, Cardiothoracovascular Department, Azienda Ospedaliero-Universitaria Careggi and University of Florence, Florence, Italy; 2 Division of Thoracic Surgery, Cardiothoracovascular Department, Azienda Ospedaliero-Universitaria Careggi and University of Florence, Florence, Italy; 3 Division of Geriatrics, Cardiothoracovascular Department, Azienda Ospedaliero-Universitaria Careggi and University of Florence, Florence, Italy; 4 Center for Atherothrombotic Disease, Cardiothoracovascular Department, Azienda Ospedaliero-Universitaria Careggi and University of Florence, Florence, Italy; 5 Department of Experimental and Clinical Medicine, University of Florence, Largo Brambilla 3, 50134 Florence, Italy

**Keywords:** Atrial fibrillation, Lung surgery, Echocardiography

## Abstract

Postoperative atrial fibrillation (PoAF) complicates 10–15% of pulmonary lobectomy and 20–30% of pneumonectomy, contributing to increased morbidity, extended hospital stays, and healthcare costs. Identifying predictors of PoAF may aid in risk stratification and preventive care. We prospectively studied 100 consecutive patients who underwent lung surgery for a malignant tumour, including video-assisted thoracic surgery (VATS) and open thoracotomy. Patients with prior atrial fibrillation, cardiac surgery, or thyroid abnormalities were excluded. All patients received pre-operative echocardiography, including speckle-tracking for left atrial (LA) and ventricular function. PoAF incidence was monitored through continuous electrocardiographic follow-up. Univariable and multivariable analyses identified clinical and echocardiographic predictors of PoAF. At univariable analysis, PoAF patients (8%) were more likely to have hypertension (100% vs. 58%, *p* = 0.018), higher fibrinogen (432 ± 118 mg/dl vs. 346 ± 87 mg/dl, *p* = 0.03), and lower magnesium levels (1.8 ± 0.2 mEq/l vs. 2.1 ± 0.2 mEq/l, *p* = 0.003). Echocardiographic differences included larger LA diameter (42 ± 5 mm vs. 35 ± 5 mm, *p* = 0.002), area (23.8 ± 3.3 cm^2^ vs. 17.7 ± 4.5 cm^2^, *p* < 0.001), and volume (36.9 ± 7.2 ml vs. 28.6 ± 9.4 ml, *p* = 0.003). Multivariable analysis identified fibrinogen (HR 1.01, *p* = 0.036), interventricular septal thickness (HR 3.05, *p* = 0.029), LA area (HR 1.33, *p* = 0.016) and LA peak contraction strain (PACS, HR 2.3, *p* = 0.023) as independent PoAF predictors. Hypertension, inflammation, electrolyte imbalance, and LA remodelling were associated with PoAF. Pre-operative identification of these factors may help target high-risk patients for preventive interventions.

## Introduction

Atrial fibrillation (AF) can be classified as either primary or secondary to other conditions, with postoperative AF (PoAF) being the most frequent secondary form [1, 2]. PoAF commonly occurs in the early postoperative period following various surgeries, particularly cardiac and non-cardiac thoracic surgeries [1]. It is a known predictor of in-hospital morbidity and short-term mortality after coronary artery bypass grafting (CABG), affecting 25–40% of patients [3]. PoAF also occurs in 10–15% of patients after pulmonary lobectomy and in 20–30% after pneumonectomy [3–5].

Whilst PoAF is initially triggered by surgery-related acute homeostatic imbalance, evidence suggests that it is not simply a transient, stand-alone event [6–9]. Instead, PoAF represents a significant surgical complication with serious prognostic implications. It is associated with increased morbidity, including a threefold increased incidence of perioperative stroke, infections, renal failure, heart failure, and myocardial infarction, as well as a 9.7% increase in mortality [10–13]. Consequently, PoAF patients often experience prolonged hospital stays and incur substantially higher healthcare costs both during and after hospitalisation.

Factors involved in the pathophysiology of PoAF include physical trauma to the atria and pericardium, local ischaemia, hypoxia, inflammation, neurohumoral activation (particularly sympathetic activation), volume redistribution, and electrolyte disturbances. Various approaches have investigated the potential predictors of PoAF, to design preventive strategies [14–16].

AF after surgery in lung cancer patients has received limited investigation, and only a few studies have focussed on possible echocardiographic predictors of PoAF in this context [10]; in the real world, pre-operative echocardiography is not mandatory for lung surgery, limiting the size and availability of retrospective studies. Pre-existing, subtle left atrial (LA) remodelling may predispose the heart’s conduction system to form re-entry circuits, which facilitate PoAF onset. Our study is aimed to evaluate structural LA abnormalities documented before surgery which may help identify patients at high risk of subsequent PoAF, with a focus on LA strain, measured via speckle-tracking echocardiography (STE). It is a quick, accessible, and sensitive parameter that quantifies LA deformation during contraction phases, reflecting LA fibrosis. Prior studies [17] have examined LA strain in various clinical scenarios; notably, peak atrial contraction strain (PACS) independently predicted AF in cryptogenic stroke patients, and both peak atrial longitudinal strain (PALS) and PACS were associated with PoAF in patients undergoing aortic stenosis and CABG surgery.

## Methods

We prospectively analysed 100 consecutive patients admitted to our department for lung cancer or metastatic nodule surgery. The primary study end-point was to determine pre-operative predictors of PoAF. The sample size was chosen arbitrarily due to feasibility and post-hoc power was calculated (data not shown).

The clinical stage was determined using whole-body computed tomography (wb-CT), positron emission tomography (PET-CT), bronchoscopy, and endoscopic techniques such as endobronchial or oesophageal ultrasound (EBUS/EUS), as well as video-mediastinoscopy. Both clinical and pathological staging were updated following the TNM classification guidelines outlined in the 8th edition by the American Joint Committee on Cancer. Patients were preferentially assigned to VATS or open thoracotomy based on tumour size and staging, location, and comorbidities. For instance, patients presenting with centrally located tumours and significant lymphadenopathy were more frequently selected for open thoracotomy due to the requisite technical considerations. Conversely, patients with smaller, peripherally located tumours, as well as those with significant comorbidities or who expressed a preference for a minimally invasive surgical approach, were assigned to video-assisted thoracoscopic surgery (VATS). This selection process aimed to optimise patient outcomes, but may have led to potential selection bias.

The surgical techniques were standardised for all cases to ensure consistent procedure performance. Protocols were established to guide the handling of vascular structures and mediastinal tissue, to reduce the risk of atrial remodelling and related postoperative complications. However, small variations were permitted based on the surgeon’s experience. The anaesthesia protocols implemented were standardised across all participants: each patient received balanced anaesthesia, initiated with propofol and remifentanil, and maintained with total intravenous anaesthesia. Comprehensive monitoring was employed throughout the procedure, which included invasive blood pressure measurement, continuous electrocardiogram monitoring, and Bispectral Index assessment to evaluate the depth of anaesthesia. Postoperative care protocols, such as pain management, early ambulation, and continuous monitoring, were consistent across patients regardless of surgical approach. However, patients who underwent VATS surgery often recovered faster and had a shorter hospital stay due to less surgical trauma.

Exclusion criteria included: age < 18 years, prior history of AF or other supraventricular arrhythmias, previous cardiac surgery, thyroid abnormalities, or unfeasible GLS analysis due to poor acoustic windows limiting LA-border tracking. Fully anonymised data were extracted from electronic hospital records. The local Ethics Committee (Regional Ethics Committee of Tuscany for Experimental Medicine, Section: Area Vasta Centro, n 21093_oss) approved the study and, under Italian observational study laws, granted a waiver of informed consent. All study procedures complied with the Declaration of Helsinki guidelines for clinical studies.

PoAF diagnosis was based on continuous electrocardiographic (ECG) monitoring during the first 4 postoperative days, in the intensive care unit (ICU) and the surgery ward. Continuous 12-lead ECG monitoring was used in the ICU setting, whilst the RootiRx system, a wearable device capturing continuous single-lead ECG data remotely, was used in the surgery ward to complete the predefined 4-day observation period. We recorded demographical, echocardiographic, pre-operative laboratory data, vital signs, surgery type, histology, and clinical course (including major and minor complications) in a dedicated database.

**Fig. 1:**
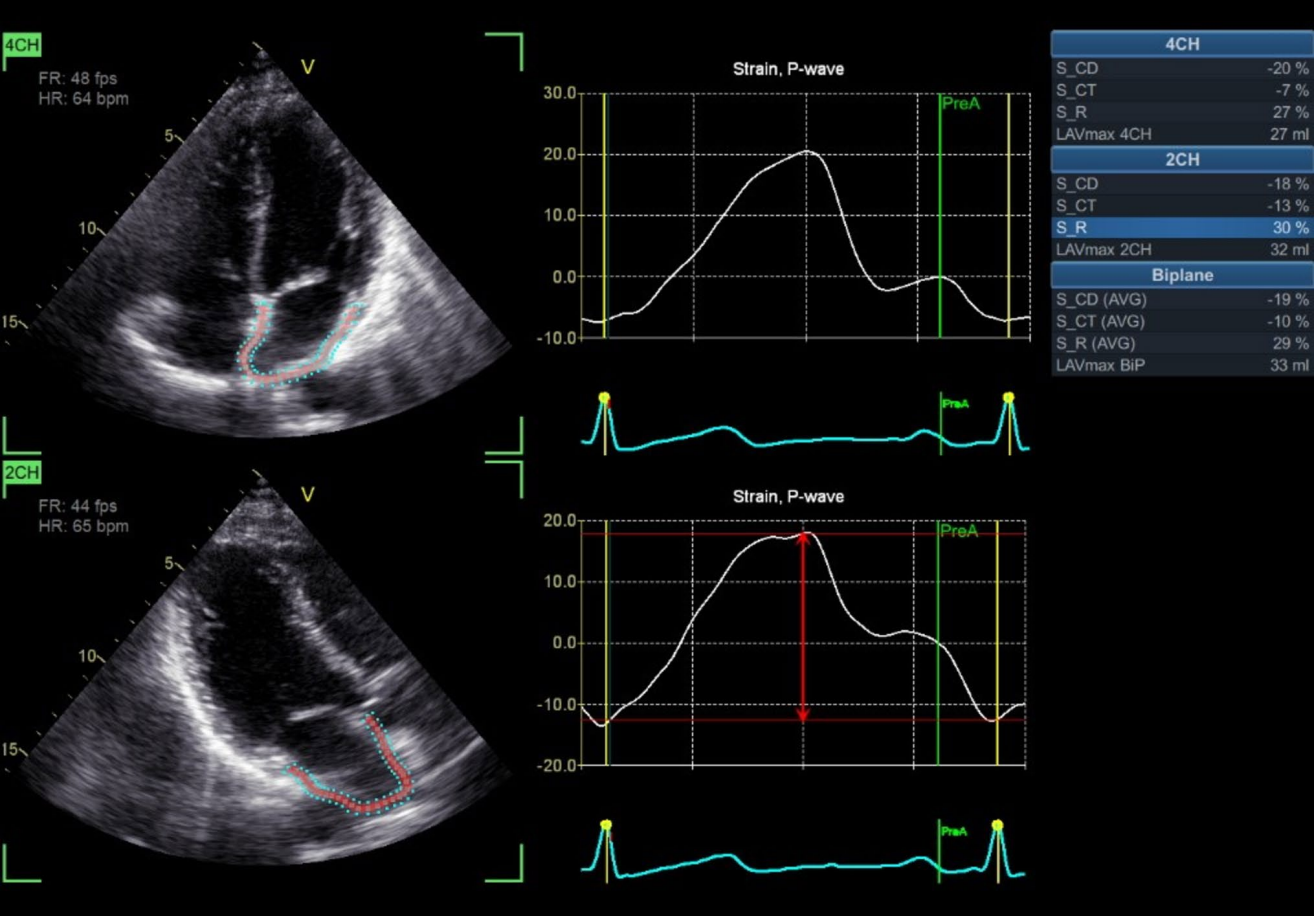
P wave (atrial end-diastole) used as zero reference point whereby first negative peak strain represents the atrial contractile function, positive peak strain corresponds to conduit function, and their sum (strain total) represents reservoir function (red arrow)

Pre-operative ECG and echocardiography were recorded in all patients, including 2D STE to measure LA and left ventricular (LV) systolic function. Echocardiograms were acquired using a General Electric VIVID E9 device and analysed with the EchoPAC Software, using a 17-segment ventricular and atrial model. For ventricular GLS analysis, we applied a conventional colour-coded map, where normal and reduced contraction are depicted as red or blue segments, respectively; atrial GLS colours were mapped in reverse. The speckle-tracking analysis utilised apical 2-, 3-, and 4-chamber views captured via 2-dimensional grayscale echocardiography with stable ECG recording. When image quality was insufficient in all three views, only the 4-chamber view was recorded. Each measurement was based on three consecutive cardiac cycles and averaged. LA strain analysis evaluated the reservoir function (in systole), conduit and contractile function (in diastole). During the reservoir phase, as the LA fills and expands, there is a positive strain that peaks at the end of the LA filling in systole, just before the mitral valve opens. Following the mitral valve’s opening, passive emptying of the left atrium begins, resulting in a decrease in atrial strain and a negative deflection in the strain curve. A second deflection in the strain curve is observed during atrial systole. Peak atrial longitudinal strain (PALS), also known as LA systolic strain, is quantified at the end of the reservoir phase. Conversely, peak atrial contraction strain (PACS), or late diastolic strain, is assessed following the P wave and corresponds to the active contraction of the atrium. We utilised a software processing method that starts at the onset of the P wave, establishing atrial end-diastole as the zero reference point. The first negative peak strain indicates atrial contractile function, whilst the positive peak strain reflects conduit function. The sum of these two measurements, known as total strain, represents reservoir function. Reference values for left atrial (LA) systolic strain, referred to as PALS, is 45.5% ± 11.4%, and the LA strain rate during late diastole, known as PACS, is − 2.11 ± 0.61 s^−1^ (see Fig. 1). Three experienced residents independently acquired echocardiographic images.

LV mass was calculated using Devereux’s formula. LV ejection fraction (LVEF, %) was calculated using the biplane method. Right ventricular systolic function was estimated using tricuspid annular plane systolic excursion (TAPSE) [18].

### Surgical technique

All surgical procedures were performed under one-lung ventilation (OLV). Muscle-sparing techniques were predominantly utilised in open procedures, such as lateral and posterolateral thoracotomy and their variations (e.g. axillary and anterior thoracotomy). These approaches enabled optimal exposure of the thoracic structures whilst minimising tissue trauma. Minimally invasive surgery was performed using uniportal and triportal VATS approaches. The uniportal technique involves a 2.5-cm incision along the anterior axillary line in the fifth or sixth intercostal space. The triportal technique, based on the Copenhagen approach, involves a 4-cm anterior incision and 2 additional smaller ports. Conversion to a muscle-sparing thoracotomy was performed when necessary to ensure surgical safety.

### Statistical analysis

Clinical variables are presented as frequency and percentage for categorical data and as mean ± SD or median (with interquartile range) for continuous data. The Chi-square test was used to compare proportions, whilst continuous variables with normal or non-normal distribution were compared with the Student’s *t* or the Mann–Whitney test, respectively. Univariable and multivariable analyses were conducted using logistic regression and general linear models; given the small size of the cohort and the low number of events, we utilised LASSO (Least Absolute Shrinkage and Selection Operator) regression to identify significant predictors of postoperative atrial fibrillation (PoAF). LASSO regression is a penalised regression technique that performs variable selection and regularisation to enhance the prediction accuracy and interpretability of the statistical model. We included in the analysis the predictors that were significant at univariable analysis and clinically relevant. We standardised the predictors before fitting the model. To determine the optimal regularisation parameter (alpha), we performed fivefold cross-validation. The alpha value that minimised the cross-validation error was selected for the final model. The model's performance was evaluated using R-squared values for the training, test, and holdout datasets.. Kaplan–Meier survival analysis and Cox regression were used to identify multivariable associations with mortality and estimate hazard ratios with 95% confidence intervals. Receiver Operating Characteristic (ROC) curve analysis was utilised to evaluate the quality of logistic regression-derived models to predict the risk of PoAF. Statistical significance was defined as a 2-sided *p* value < 0.05. Analyses were conducted with SPSS (version 27) statistical package.

## Results

Amongst the 100 patients, 74% received VATS, whilst 26% underwent open surgery. The procedures included 56 lobectomies (57%), 22 segmentectomies (22%), 18 atypical resections (18%), 1 pneumonectomy (1%), and 2 sleeve bronchial resections (2%). The type of surgery was not associated with PoAF. Intra-operative complications (bleeding) were reported in two patients; one developed PoAF.

**Fig. 2:**
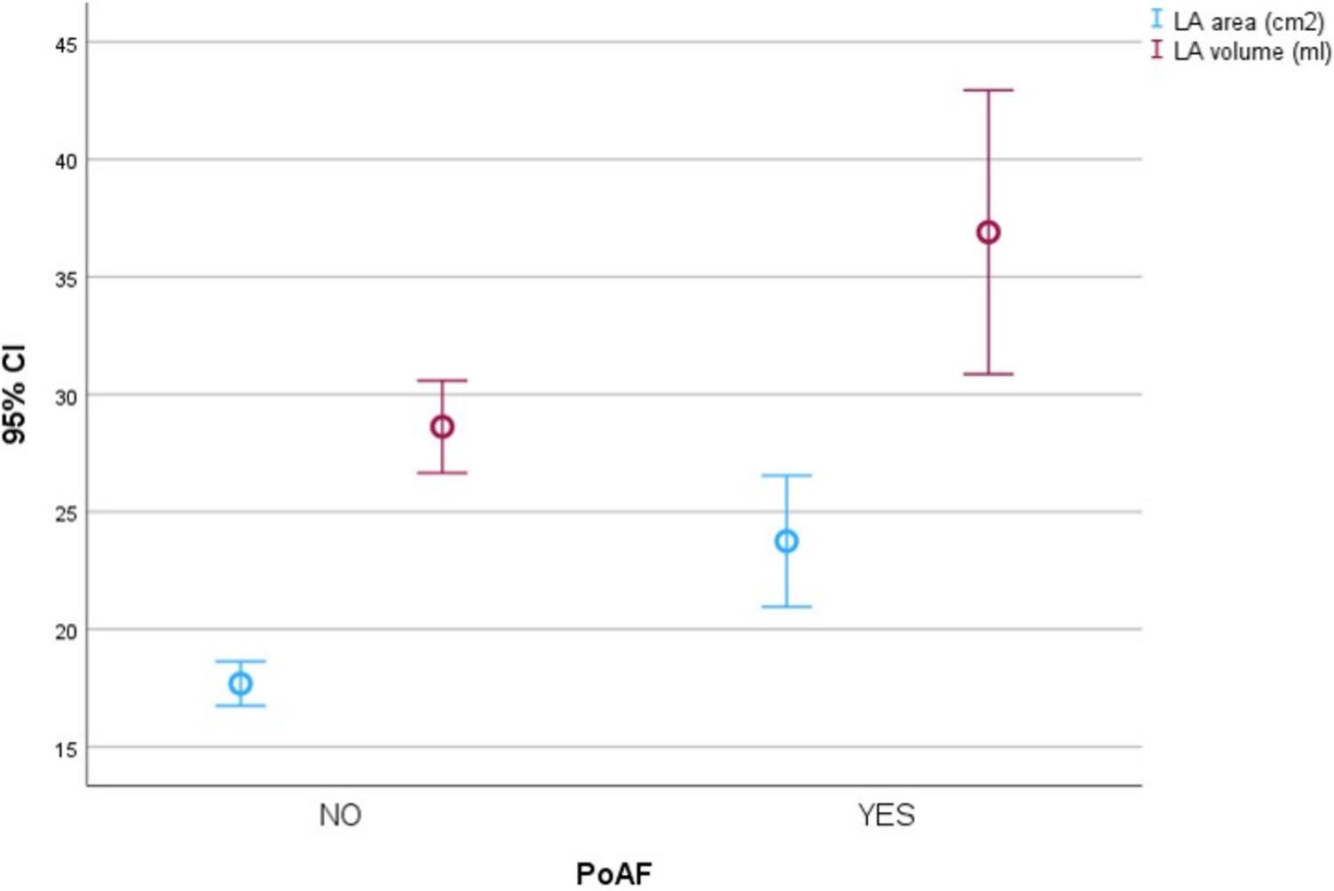
Left atrial area and volume by PoAF occurrence (mean and 95% CI)

Out of 100 study patients, 8 developed PoAF; of these, 6 were asymptomatic and with self-limited PoAF (minimal and maximal duration 28 and 213 min, respectively), whilst two were symptomatic and had a long-lasting PoAF that required successful pharmacological cardioversion with i.v. amiodarone. Those two patients were prescribed oral anticoagulation due to a high CHA_2_DS_2_-VASc score (Table 1). Therefore, all PoAF patients were discharged in sinus rhythm (2 on anticoagulant therapy). Table 1 shows the results of the univariable analysis including the demographical, laboratory, echocardiographic, histological characteristics and outcomes of 100 patients undergoing lung surgery for malignancy, by PoAF incidence. Patients who developed PoAF showed several distinct characteristics compared to those who did not. Despite PoAF being more frequent in older males, sex distribution and age were not significantly different between the two groups, as a probable consequence of the small sample size. Moreover, exclusion criteria (prior history of AF or other supraventricular arrhythmias, previous cardiac surgery, thyroid abnormalities, or unfeasible GLS analysis due to poor acoustic windows) selected patients with a low probability of cardiac disease, which is more frequent in male and older patients. The prevalence of hypertension, however, was significantly higher in the PoAF group. Pharmacological treatment before surgery was similar in the two groups (data not shown) and no patient received prophylactic anti-arrhythmic agents. Notable laboratory differences included higher fibrinogen and lower magnesium levels in PoAF patients, suggesting a pathophysiological impact for both inflammation and electrolyte imbalance. The prevalence of primitive (vs. metastatic) malignancy, and the histological type of lung cancer, were similar in the two PoAF groups. Echocardiographic differences were numerous and remarkable. Consistently with the higher prevalence of hypertension, PoAF patients had larger wall thickness, volumes and ventricular mass, whereas LV systolic function was normal in both groups. All anatomical and functional left atrial (LA) data consistently indicated that PoAF patients had a remarkable degree of LA remodelling (Fig. 2) and systolic dysfunction. On the other hand, all the operative and postoperative surgery data were similar in the two groups. In-hospital, surgery-related events (pneumo- and/or haemothorax) were alike in the two groups, on the contrary PoAF was associated with a prolonged hospital stay, suggesting a higher healthcare burden in these patients. All patients were discharged home.

**Table 1:** Univariable analysis of the demographical, laboratory, histological, echocardiographic characteristics, and outcomes of 100 patients undergoing lung surgery for malignancy, by occurrence of PoAF

Covariate	No PoAF (*N* = 92)	PoAF (*N* = 8)	*p* value
*Demographical variables*			
Females, *N* (%)	47 (51%)	2 (25%)	0.15
Age, years, mean ± SD	67 ± 10	73 ± 4	0.14
Previous or current smoking, *N* (%)	70 (76%)	7 (88%)	0.49
Hypertension, *N* (%)	53 (58%)	8 (100%)	0.018
Diabetes, *N* (%)	15 (16%)	3 (37%)	0.13
Chronic kidney disease, *N* (%)	23 (25%)	2 (25%)	0.63
Coronary artery disease, *N* (%)	5 (5%)	1 (12%)	0.42
CHA_2_DS_2_-VASc score, mean ± SD	2.3 ± 1.2	3 ± 0.9	0.82
*Pre-operative clinical and laboratory parameters*			
SO_2_, mean ± SD	97 ± 2	96 ± 2	0.42
WBC × 10^3^, u/µl, mean ± SD	10 ± 25	10 ± 6	0.42
Hb, g/dl, mean ± SD	12.9 ± 1.6	12.6 ± 2.4	0.37
Platelets × 10^3^, u/µl, mean ± SD	237 ± 67	264 ± 67	0.16
Fibrinogen, mg/dl, mean ± SD	346 ± 87	432 ± 118	0.03
TSH, U/µl, mean ± SD	2.4 ± 2	1.7 ± 0.8	0.52
Creatinine, mg/dl, mean ± SD	0.84 ± 0.23	0.89 ± 0.22	0.45
Serum potassium, mEq/l, mean ± SD	4.4 ± 0.4	4.3 ± 0.5	0.86
Serum magnesium, mEq/l, mean ± SD	2.1 ± 0.2	1.8 ± 0.2	0.003
*Histological data*			
Primitive lung cancer, *N* (%)	82 (89%)	6 (75%)	0.32
Histology			
Adenocarcinoma, *N* (%)	48 (52%)	4 (50%)	0.94
Squamous cell carcinoma, *N* (%)	15 (16%)	2 (25%)	
*Echocardiographic variables*			
IVS, mm, mean ± SD	9 ± 1	10 ± 1	0.011
PW, mm, mean ± SD	9 ± 4	10 ± 1	0.01
LVMI, g/m^2^, mean ± SD	74.16 ± 14.9	93 ± 23.7	0.035
EDV, ml, mean ± SD	91 ± 23	111 ± 22	0.024
ESV, ml, mean ± SD	33 ± 11	42 ± 9	0.023
LVEF, %, mean ± SD	64 ± 7	62 ± 5	0.34
TAPSE, mm, mean ± SD	22 ± 3	23 ± 2	0.52
S′, cm/s, mean ± SD	12.4 ± 1.9	12.1 ± 1.2	0.86
RV/RA gradient, mm Hg, mean ± SD	18.8 ± 7.1	23.4 ± 8.0	0.11
E/A, mean ± SD	0.89 ± 0.28	0.98 ± 0.3	0.35
Left atrial diameter, mm, mean ± SD	35 ± 5	42 ± 5	0.002
Left atrial area, cm^2^, mean ± SD	17.7 ± 4.5	23.8 ± 3.3	< 0.001
Left atrial volume, ml, mean ± SD	28.6 ± 9.4	36.9 ± 7.2	0.003
Peak left atrial contraction strain (PACS), mean ± SD	− 2.55 ± 2.68	− 1.1 ± 2.23	0.014
Peak left atrial longitudinal strain (PALS), mean ± SD	29 ± 6	25 ± 10	0.3
Left ventricular global longitudinal strain, mean ± SD	− 18.4 ± 2.7	− 17.8 ± 1.6	0.38
*Operative and perioperative details*			
Pericardial injury, *N* (%)	16 (17.6%)	2 (25%)	0.6
Video-assisted thoracic surgery, *N* (%)	70 (76%)	4 (50%)	0.15
Left thoracic access, *N* (%)	38 (41%)	5 (62%)	0.24
Operative duration, minutes, mean ± SD	135 ± 54	165 ± 57	0.12
Post-surgical transfusion, *N* (%)	38 (41%)	2 (25%)	0.36
Post-surgical bronchoscopy, *N* (%)	8 (9%)	2 (25%)	0.14
In-hospital events, *N* (%)	6 (6.5%)	1 (12.5%)	0.45
Length of hospital stay, days, mean ± SD	7.4 ± 8	12.0 ± 9	0.033

*WBC* white blood cells, *TSH* thyroid stimulating hormone, *IVS* interventricular septum, *PW* posterior wall, *LVMI* left ventricle mass index, *EDV* end-diastolic volume, *ESV* endo systolic volume, *LVEF* left ventricular ejection fraction, *TAPSE* tricuspid annular plane systolic excursion, *RV/RA* right ventricle/right atrium

At multivariable analysis using binary logistic regression with all univariable significant parameters, fibrinogen levels (HR 1.01, 95% CI 1.00–1.02, *p* = 0.036), IVS thickness (HR 3.05, 95% CI 1.12–8.26, *p* = 0.029), LA area (HR 1.33, 95% CI 1.06–1.68, *p* = 0.016), and PACS (HR 2.3, 95% CI 1.12–5.02, *p* = 0.023), were independently associated with the risk of PoAF. Even in a multivariable analysis including exclusively the surgical access (VATS vs. open surgery) and atrial dimensions, LA area resulted in being an independent predictor of PoAF (HR 1.25, 95% CI 1.08–1.46, *p* = 0.003), confirming to be a risk factor for PoAF independently from the type of surgery. Given the low number of events and the risk of overfitting, we performed a LASSO analysis including the following predictors: hypertension, fibrinogen, magnesium, LA area, LA volume, and PACS. The LASSO regression analysis identified several predictors with non-zero coefficients, indicating their importance in predicting PoAF. The optimal alpha value selected through cross-validation was 0.01. The model explained 33.4% of the variance in the training data (*R*-squared = 0.334). However, the model’s performance on the test data was lower, with an average *R*-squared of 0.14. The holdout *R*-squared was − 0.54, suggesting poor generalisation to unseen data.

The regression coefficients for the selected predictors are reported in Table 2.

**Table 2:** LASSO (least absolute shrinkage and selection operator) regression analysis

Covariate	Standardised regression coefficient
Hypertension	0.059
Fibrinogen	0.001
Magnesium	− 0.221
Left atrial area	0.020
Left atrial volume	0.001
Peak left atrial contraction strain	0.006

**Fig. 3:**
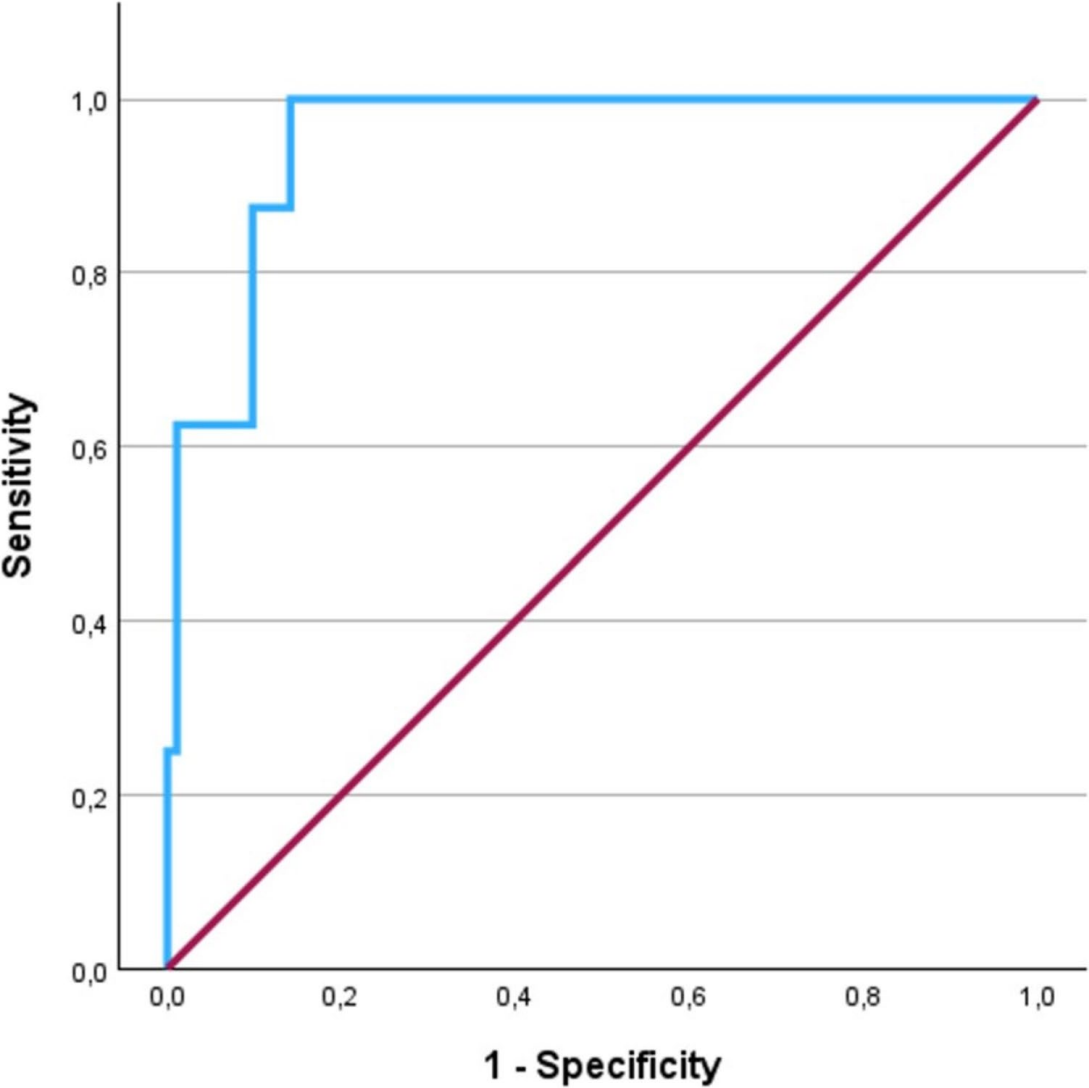
ROC-derived curve of the multivariable model for prediction of PoAF

The ROC-derived AUC of the multivariable model was 0.95 (Fig. 3).

## Discussion

The occurrence of PoAF is associated with a fourfold increased risk of stroke and a 3.5-fold higher long-term mortality during follow-up, indicating not only the presence of comorbidities but also predicting the likelihood of cardiovascular events [19]. Consequently, identifying PoAF predisposing factors is critical for recognising at-risk patients, who may benefit from enhanced rhythm monitoring and personalised clinical interventions to improve long-term postoperative outcomes. Nonetheless, the existing literature remains insufficient regarding risk factors for AF following thoracic surgery.

This study examines the predictive factors for PoAF in patients undergoing elective lung surgery for malignancies, revealing key associations with several indicators of pre-existing LA remodelling and systemic inflammation. In the univariable analysis of our cohort, PoAF patients exhibited significantly larger LA dimensions, increased fibrinogen levels, lower magnesium levels, and greater LV mass and volumes, amongst other clinical markers. These findings are largely consistent with previous literature on PoAF across various surgical contexts, yet they also underscore specific nuances associated with lung surgery, a relatively understudied population in this field.

The prevalence of hypertension amongst PoAF patients in our study aligns with extensive evidence highlighting hypertension as a consistent risk factor for PoAF in cardiac and thoracic surgery. In particular, hypertension’s role in promoting LA remodelling and structural changes is well-documented, as noted by other studies in the context of CABG surgery, where hypertension correlates with increased LA pressure and wall stress, predisposing patients to arrhythmias [20]. Our finding that 100% of PoAF patients had hypertension supports the relevance of hypertensive atrial changes in this population, which merit further pre-operative screening and management.

Our study also found significantly elevated fibrinogen levels in PoAF patients, reflecting a potential role of systemic inflammation in increasing PoAF risk. Elevated fibrinogen has previously been implicated in PoAF development after cardiac surgery, with Mesen et al. suggesting that systemic inflammation can accelerate atrial fibrosis and remodelling, facilitating AF onset [21]. This association between inflammation and PoAF underscores the potential benefit of exploring anti-inflammatory strategies or therapies to reduce fibrinogen levels as preventive measures in high-risk patients.

Reduced magnesium was another significant finding associated with PoAF in the univariable analysis of our study, supporting earlier research linking electrolyte imbalances to arrhythmogenesis. Low-magnesium levels can increase myocardial excitability and susceptibility to re-entrant circuits, both conducive to AF development. This finding further supports the importance of postoperative monitoring and correcting magnesium levels to potentially reduce PoAF incidence. Previous studies have shown that magnesium supplementation can effectively decrease PoAF rates in cardiac surgery [22], and others are ongoing [23], suggesting similar interventions might benefit lung surgery patients at risk for arrhythmias.

Echocardiographic analysis of LA structure and function revealed substantial differences between PoAF and non-PoAF patients in our study. Specifically, patients who developed PoAF exhibited significantly larger LA diameters, areas, and volume, along with lower LA contraction strain, all indicative of structural and functional remodelling. Prior research by Yuda et al. highlighted that impaired LA strain predicts AF risk in several clinical populations, and similar associations have been identified in cardiac surgery [24]. Our findings contribute to this body of evidence by highlighting the relevance of pre-operative LA strain analysis in predicting PoAF in lung surgery. Importantly, in multivariable analysis, we found that LA area and PACS were independent predictors of PoAF. These parameters could be non-invasive markers for identifying high-risk individuals and tailoring perioperative care. Although lower in patients with PoAF, PALS values did not reach statistical significance, probably due to the small sample size.

Higher LV mass index and increased LV end-diastolic and end-systolic volumes were also associated with PoAF in our study. These associations are consistent with findings from previous studies, such as those by Xiang et al., where LV hypertrophy and diastolic dysfunction correlated with increased LA pressure and volume, creating a substrate favourable for AF [25].

The LASSO regression analysis identified fibrinogen, magnesium, LA area, LA volume, and PACS as significant predictors of PoAF. Despite the model explaining a moderate variance in the training data, its performance on the test and holdout datasets was suboptimal. This could be due to the small sample size. Future research should focus on refining the model in larger, multicentric cohorts. In addition,, further validation with independent datasets is necessary to confirm the model's robustness and generalizability. We believe that our results can be the first step in exploring this complex setting. LA structural and functional pre-operative assessment might become the crucial evaluation for identifying patients at high risk of PoAF before cancer lung surgery.

Our results align with the latest research on atrial cardiomyopathy [26]. Patients who developed PoAF exhibited significant abnormalities in atrial structure (i.e. increased diameters, area, and volume) and function (i.e. reduced LA longitudinal strain). Furthermore, these patients displayed elevated bio-humoral markers associated with systemic inflammation. Consequently, it appears that thoracic surgery may have served as a trigger for PoAF, particularly in individuals with vulnerable atrial tissue and a predisposed bio-humoral profile. Furthermore, we observed that PoAF patients experienced prolonged hospital stays, corroborating reports from other studies that associated PoAF with greater healthcare resource utilisation and higher healthcare costs. This reinforces the importance of early identification and preventive strategies to mitigate the complications and economic burden of PoAF.

The majority of patients with PoAF were asymptomatic, which raises the question of whether they might experience additional asymptomatic episodes of atrial fibrillation (AF). Therefore, the potential for post-discharge rhythm monitoring using consumer-led screening tools, such as handheld ECG devices, becomes particularly relevant. This approach may help detect AF, which is increasingly prevalent and could facilitate the integration of guideline-approved therapeutic regimens and oral anticoagulation [27]. Moreover, implementing such technologies in telemedicine and virtual clinics is already feasible. However, the accuracy and reliability of wearable devices and the interpretation of the data they generate remain topics of ongoing debate and require further validation [28].

Dealing with stroke risk and prevention, PoAF patients had an average CHA_2_DS_2_-VASc score of 3 ± 0.9, however, only two out of eight received oral anticoagulation. The decision to initiate anticoagulation was based on the duration of the arrhythmic episodes, regardless of whether patients were symptomatic, as asymptomatic status should not be used as a rationale against anticoagulation [29]. Despite new guidelines recommending anticoagulation in PoAF patients with a class IIa indication [1], there are concerns regarding the potential risk of bleeding when initiating anticoagulation therapy during the post-surgical period. Indeed, although tools like the HAS-BLED and DOAC-score can predict bleeding risk, their effectiveness is modest [30] and has not been validated specifically for patients undergoing thoracic surgery, which raises further questions about their applicability in this context. Additional clinical data are needed to provide better guidance on anticoagulant prescription following thoracic surgery.

## Limitations and future directions

Our study’s limitations include its single-centre, observational design and the relatively small sample size, which may affect the generalizability of our findings. Second, the criteria for allocating patients to each surgical technique to optimise surgical outcomes may have introduced potential selection bias. In addition, the dependence on image quality in a population with classically difficult acoustic windows may imply a time-consuming procedure, which may prevent the widespread clinical application of the assessment of LA function. However, since our images were acquired by residents, though experienced ones, this excludes the necessity of long-lasting formation. Future studies with larger, multicentre cohorts are warranted to validate our findings further and explore the role of targeted interventions, such as routine administration of magnesium or anti-inflammatory agents to high-risk patients, in reducing PoAF incidence. In conclusion, our findings suggest that elevated fibrinogen and echocardiographic markers of LA remodelling and dysfunction might be valuable predictors of PoAF in lung cancer surgery. Future research should focus on refining these predictive models and investigating potential interventions tailored to lung surgery patients at high risk for PoAF. Furthermore, additional data are essential to elucidate the long-term implications of PoAF, which has been correlated with an elevated risk of long-term stroke and mortality [31].

## Data Availability

Data and material are available on a reasonable request from the author.

## References

[1] Van Gelder IC, Rienstra M, Bunting KV, Casado-Arroyo R, Caso V, Crijns HJGM, De Potter TJR, Dwight J, Guasti L, Hanke T, Jaarsma T, Lettino M, Løchen ML, Lumbers RT, Maesen B, Mølgaard I, Rosano GMC, Sanders P, Schnabel RB, Suwalski P, Svennberg E, Tamargo J, Tica O, Traykov V, Tzeis S, Kotecha D, ESC Scientific Document Group (2024) 2024 ESC guidelines for the management of atrial fibrillation developed in collaboration with the European Association for Cardio-Thoracic Surgery (EACTS). Eur Heart J 45(36):3314–3414. 10.1093/eurheartj/ehae176 39210723 10.1093/eurheartj/ehae176

[2] Bandyopadhyay D, Ball S, Hajra A, Chakraborty S, Dey AK, Ghosh RK, Palazzo AM (2019) Impact of atrial fibrillation in patients with lung cancer: insights from National Inpatient Sample. Int J Cardiol Heart Vasc 22:216–217 30963100 10.1016/j.ijcha.2019.02.012 PMC6437280

[3] Semeraro GC, Meroni CA, Cipolla CM, Cardinale DM (2021) Atrial fibrillation after lung cancer surgery: prediction, prevention and anticoagulation management. Cancers (Basel) 13(16):4012 34439166 10.3390/cancers13164012 PMC8394120

[4] Yun JP, Choi EK, Han KD, Park SH, Jung JH, Park SH, Ahn HJ, Lim JH, Lee SR, Oh S (2021) Risk of atrial fibrillation according to cancer type: a nationwide population-based study. JACC CardioOncol 3(2):221–232 34396327 10.1016/j.jaccao.2021.03.006 PMC8352078

[5] Jakobsen CB, Lamberts M, Carlson N, Lock-Hansen M, Torp-Pedersen C, Gislason GH, Schou M (2019) Incidence of atrial fibrillation in different major cancer subtypes: a Nationwide population-based 12 year follow up study. BMC Cancer 19(1):1105 31726997 10.1186/s12885-019-6314-9 PMC6854796

[6] Roselli EE, Murthy SC, Rice TW, Houghtaling PL, Pierce CD, Karchmer DP, Blackstone EH (2005) Atrial fibrillation complicating lung cancer resection. J Thorac Cardiovasc Surg 130(2):438–444 16077410 10.1016/j.jtcvs.2005.02.010

[7] Bagheri R, Yousefi Y, Rezai R, Azemonfar V, Keshtan FG (2019) Atrial fibrillation after lung surgery: incidence, underlying factors, and predictors. Kardiochir Torakochirurgia Pol 16(2):53–56 31410090 10.5114/kitp.2019.86355 PMC6690155

[8] Garner M, Routledge T, King JE, Pilling JE, Veres L, Harrison-Phipps K, Bille A, Harling L (2017) New-onset atrial fibrillation after anatomic lung resection: predictive factors, treatment and follow-up in a UK thoracic centre. Interact Cardiovasc Thorac Surg 24(2):260–264 27803121 10.1093/icvts/ivw348

[9] Onaitis M, D’Amico T, Zhao Y, O’Brien S, Harpole D (2010) Risk factors for atrial fibrillation after lung cancer surgery: analysis of the Society of Thoracic Surgeons general thoracic surgery database. Ann Thorac Surg 90(2):368–374 20667313 10.1016/j.athoracsur.2010.03.100

[10] He G, Yao T, Zhao L, Geng H, Ji Q, Zuo K, Luo Y (2020) Atrial fibrillation and alteration of heart rate variability after video-assisted pulmonary lobectomy versus thoracotomy pulmonary lobectomy. J Cardiothorac Surg 15(1):220 32795332 10.1186/s13019-020-01260-6 PMC7427877

[11] Yao C, Veleva T, Scott L Jr, Cao S, Li L, Chen G, Jeyabal P, Pan X, Alsina KM, Abu-Taha I, Ghezelbash S, Reynolds CL, Shen YH, LeMaire SA, Schmitz W, Müller FU, El-Armouche A, Tony Eissa N, Beeton C, Nattel S, Wehrens XHT, Dobrev D, Li N (2018) Enhanced cardiomyocyte NLRP3 inflammasome signaling promotes atrial fibrillation. Circulation 138(20):2227–2242 29802206 10.1161/CIRCULATIONAHA.118.035202 PMC6252285

[12] Wang H, Wang Z, Zhou M, Chen J, Yao F, Zhao L, He B (2021) Postoperative atrial fibrillation in pneumonectomy for primary lung cancer. J Thorac Dis 13(2):789–802 33717552 10.21037/jtd-20-1717 PMC7947480

[13] Ishibashi H, Wakejima R, Asakawa A, Baba S, Nakashima Y, Seto K, Kobayashi M, Okubo K (2020) Postoperative atrial fibrillation in lung cancer lobectomy-analysis of risk factors and prognosis. World J Surg 44(11):3952–3959 32681318 10.1007/s00268-020-05694-w

[14] Gong J, Wang X, Liu Z, Yao S, Xiao Z, Zhang M, Zhang Z (2021) Risk factors and survival analysis of arrhythmia following lung cancer surgery: a retrospective study. J Thorac Dis 13(2):847–860 33717558 10.21037/jtd-20-2740 PMC7947489

[15] Kavurmaci O, Akcam TI, Ergonul AG, Turhan K, Cakan A, Cagirici U (2018) Is the risk of postoperative atrial fibrillation predictable in patients undergoing surgery due to primary lung cancer? Heart Lung Circ 27(7):835–841 28800934 10.1016/j.hlc.2017.06.729

[16] Elahi MM, Flatman S, Matata BM (2008) Tracing the origins of postoperative atrial fibrillation: the concept of oxidative stress-mediated myocardial injury phenomenon. Eur J Cardiovasc Prev Rehabil 15(6):735–741 19020458 10.1097/HJR.0b013e328317f38a

[17] Pastore MC, Degiovanni A, Grisafi L, Renda G, Sozzani M, Giordano A, Salvatici C, Lorenz V, Pierfelice F, Cappelli C, De Donno F, Focardi M, Ricci F, Benedetto U, Gallina S, Cameli M, Patti G (2024) Left atrial strain to predict postoperative atrial fibrillation in patients undergoing coronary artery bypass grafting. Circ Cardiovasc Imaging 17(1):e015969 38227692 10.1161/CIRCIMAGING.123.015969

[18] Echahidi N, Pibarot P, O’Hara G, Mathieu P (2008) Mechanisms, prevention, and treatment of atrial fibrillation after cardiac surgery. J Am Coll Cardiol 51(8):793–801 18294562 10.1016/j.jacc.2007.10.043

[19] Albini A et al (2021) Long-term outcomes of postoperative atrial fibrillation following non cardiac surgery: a systematic review and metanalysis. Eur J Intern Med 85:27–33. 10.1016/j.ejim.2020.12.018 33402281 10.1016/j.ejim.2020.12.018

[20] Mitchell C, Rahko PS, Blauwet LA, Canaday B, Finstuen JA, Foster MC, Horton K, Ogunyankin KO, Palma RA, Velazquez EJ (2019) Guidelines for performing a comprehensive transthoracic echocardiographic examination in adults: recommendations from the American Society of Echocardiography. J Am Soc Echocardiogr 32(1):1–64 30282592 10.1016/j.echo.2018.06.004

[21] Maesen B, Nijs J, Maessen J, Allessie M, Schotten U (2012) Post-operative atrial fibrillation: a maze of mechanisms. Europace 14(2):159–174 21821851 10.1093/europace/eur208 PMC3262403

[22] Chaudhary R, Garg J, Turagam M, Chaudhary R, Gupta R, Nazir T, Bozorgnia B, Albert C, Lakkireddy D (2019) Role of prophylactic magnesium supplementation in prevention of postoperative atrial fibrillation in patients undergoing coronary artery bypass grafting: a systematic review and meta-analysis of 20 randomized controlled trials. J Atrial Fibrillation 12(1):2154 10.4022/jafib.2154 PMC6811340 31687067

[23] Meerman M, Buijser M, van den Berg L, van den Heuvel AM, Hoohenkerk G, van Driel V, Munsterman L, de Vroege R, Bailey M, Bellomo R, Ludikhuize J (2024) Magnesium sulphate to prevent perioperative atrial fibrillation in cardiac surgery: a randomized clinical trial: a protocol description of the PeriOperative Magnesium Infusion to Prevent Atrial fibrillation Evaluated (POMPAE) trial. Trials 25(1):540 39148128 10.1186/s13063-024-08368-3 PMC11328354

[24] Yuda S, Muranaka A, Miura T (2016) Clinical implications of left atrial function assessed by speckle tracking echocardiography. J Echocardiogr 14(3):104–112 26951561 10.1007/s12574-016-0283-7

[25] Xiang H, Xue Y, Chen Z, Yu Y, Peng Y, Wang J, Ji K, Zhu H (2021) The association between left ventricular hypertrophy and the occurrence and prognosis of atrial fibrillation: a meta-analysis. Front Cardiovasc Med 30(8):639993 10.3389/fcvm.2021.639993 PMC8362884 34395549

[26] Goette A, Corradi D, Dobrev D et al (2024) Atrial cardiomyopathy revisited-evolution of a concept: a clinical consensus statement of the European Heart Rhythm Association (EHRA) of the ESC, the Heart Rhythm Society (HRS), the Asian Pacific Heart Rhythm Society (APHRS), and the Latin American Heart Rhythm Society (LAHRS). Europace 26(9):204. 10.1093/europace/euae204 10.1093/europace/euae204 PMC11431804 39077825

[27] Brandes A, Stavrakis S, Freedman B et al (2022) Consumer-led screening for atrial fibrillation: frontier review of the AF-SCREEN International Collaboration. Circulation 146(19):1461–1474. 10.1161/CIRCULATIONAHA.121.058911 36343103 10.1161/CIRCULATIONAHA.121.058911 PMC9673231

[28] Svennberg E, Caiani EG, Bruining N et al (2023) The digital journey: 25 years of digital development in electrophysiology from an Europace perspective. Europace 25(8):euad176. 10.1093/europace/euad176 37622574 10.1093/europace/euad176 PMC10450797

[29] Lip GYH, Proietti M, Potpara T et al (2023) Atrial fibrillation and stroke prevention: 25 years of research at *EP Europace Journal*. Europace 25(9):euad226. 10.1093/europace/euad226 37622590 10.1093/europace/euad226 PMC10451006

[30] Mei DA, Imberti JF, Bonini N et al (2024) Performance of HAS-BLED and DOAC scores to predict major bleeding events in atrial fibrillation patients treated with direct oral anticoagulants: a report from a prospective European observational registry. Eur J Intern Med 128:63–70. 10.1016/j.ejim.2024.06.022 38969571 10.1016/j.ejim.2024.06.022

[31] Lin MH, Kamel H, Singer DE, Wu YL, Lee M, Ovbiagele B (2019) Perioperative/postoperative atrial fibrillation and risk of subsequent stroke and/or mortality. Stroke 50(6):1364–1371. 10.1161/STROKEAHA.118.023921 31043148 10.1161/STROKEAHA.118.023921

